# Centurial Variation in Size at Maturity of Eastern Baltic Cod (*Gadus morhua*) Mirrors Conditions for Growth

**DOI:** 10.1002/ece3.70382

**Published:** 2024-10-13

**Authors:** Henrik Svedäng, Sara Hornborg, Anders Grimvall

**Affiliations:** ^1^ Baltic Sea Centre Stockholm University Stockholm Sweden; ^2^ Swedish Institute for the Marine Environment (SIME) Gothenburg University Gothenburg Sweden; ^3^ Department Agriculture and Food RISE Research Institutes of Sweden Gothenburg Sweden

**Keywords:** Baltic cod, ecosystem functioning, fishery‐induced evolution, growth potential, size at maturity, stock productivity

## Abstract

The status of Eastern Baltic cod (EBC) *Gadus morhua* has remained poor despite low fishing mortality for over a decade, including a fishing ban since 2019. Although the decline in productivity can be explained by lower individual growth and survival rates, other aspects of life‐history changes such as maturation patterns for EBC has so far not been sufficiently explored. According to current stock assessments, the median size at maturity (*L*_50_) has halved from 40 to around 20 cm in total length since the 1990s, while the overall size distribution has become increasingly truncated. It has previously been suggested that changes in *L*_50_ can be attributed to both fishing‐induced evolution and phenotypic plasticity induced by growth rates. However, since *L*_50_ is currently occurring around 20 cm, the maturation process must have been initiated at much smaller sizes, that is, long before the fish could be caught in the dominant trawl fishery at around 35 cm. In this study, we aimed to further investigate what drivers may have led to reduced productivity in EBC by determining variations in size at sexual maturity in longer time series than has been done before (1930s to 1980s) and include prey productivity and quality. We found that *L*_50_ declined already in the 1930s and thereafter remained stable at around 40 cm up to the 1990s. On a centurial perspective, *L*_50_ has been positively correlated to growth potential (*L*_95_), length diversity, total stock biomass, total catch and yield per recruit, while Fulton's condition factor was not related to *L*_50_. Our results suggest that the links between life‐history parameters and external drivers are complex, but the present unprecedented early onset of maturity and hence decline in *L*_50_ since the 1990s signals a decline in growth potential, which also has hampered the productivity of EBC.

## Graphical abstract

The status of Eastern Baltic cod (EBC) *Gadus morhua* has remained poor despite low fishing mortality for over a decade, including a fishing ban since 2019. Our results suggest that the links between life‐history parameters and external drivers are complex, but the present unprecedented reduced size at maturity since the 1990s signals a decline in growth potential, which also has hampered the productivity of EBC.

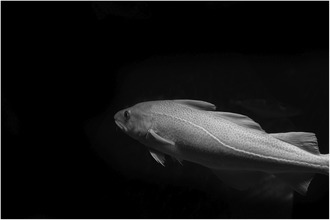

## 1 Introduction

### 1.1 The Eastern Baltic Cod Stock

**FIGURE 1:**
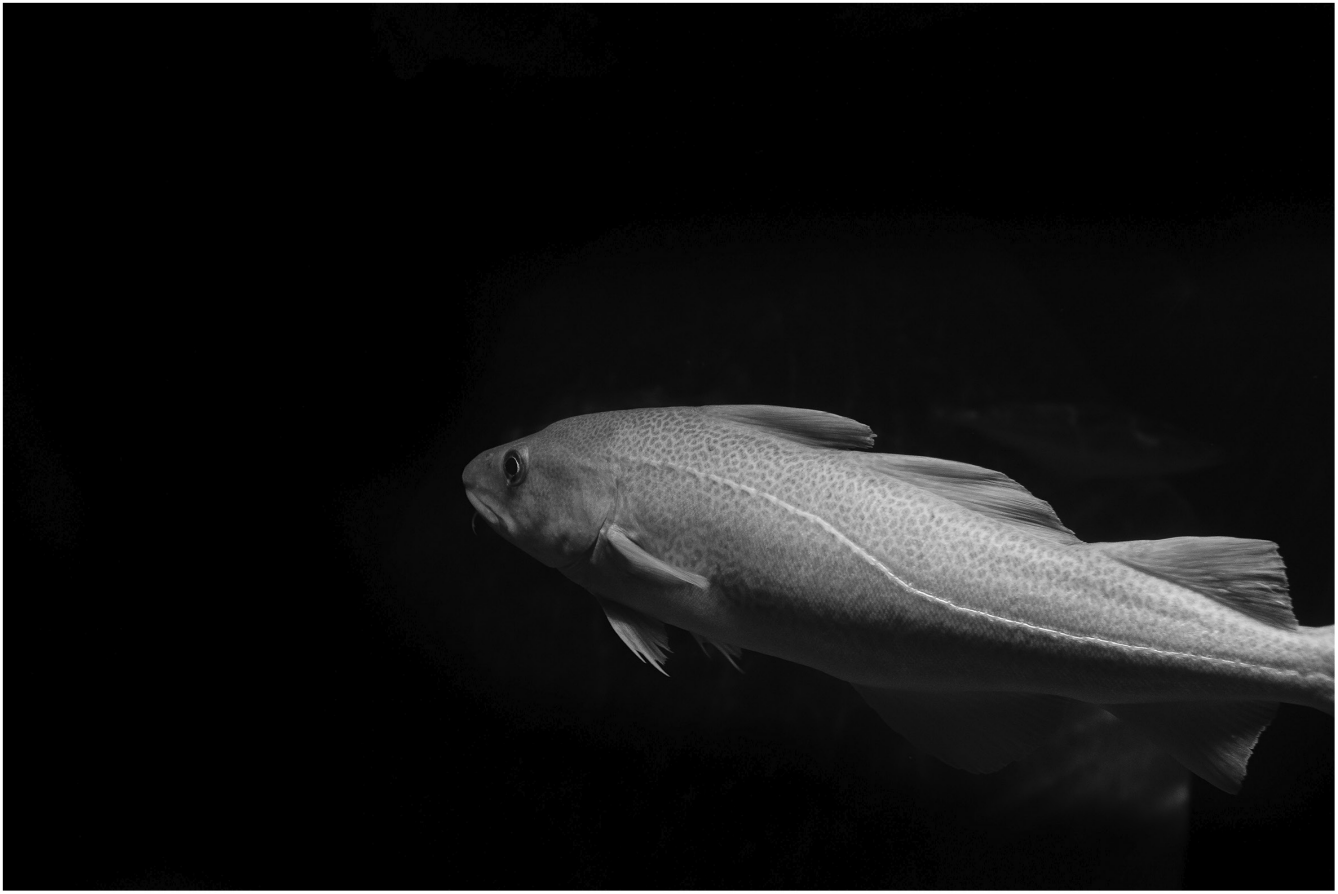
A swimming Baltic cod (*Gadus morhua*). Photo: Ricardo Resende/Unsplash.

**FIGURE 2:**
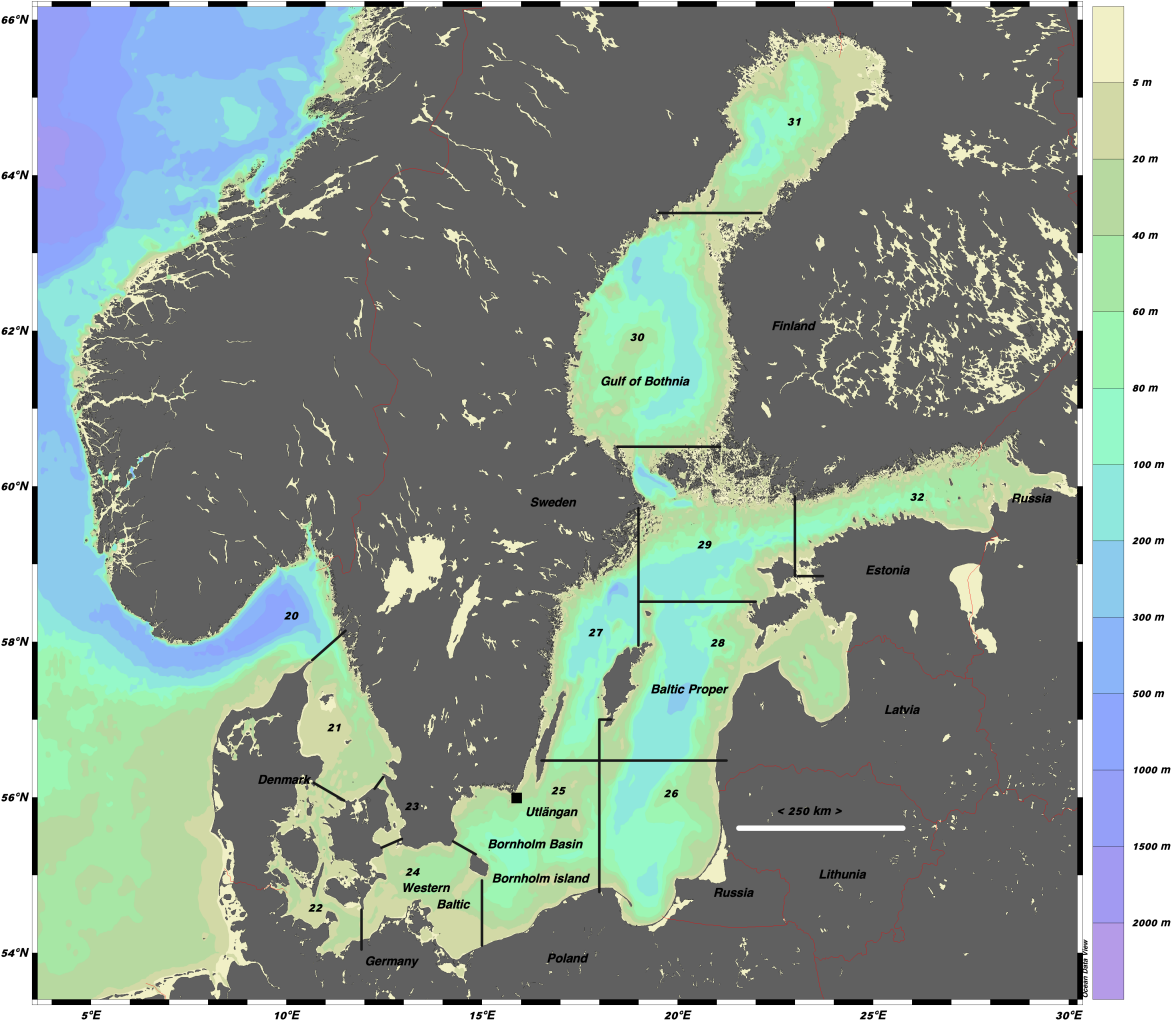
The Baltic Sea. The borders and numbers of the ICES subdivisions (SD) are as defined by the International Council for the Exploration of the Sea (www.ices.dk). The black square indicates where the herring samples were taken for fat analysis.

**FIGURE 3:**
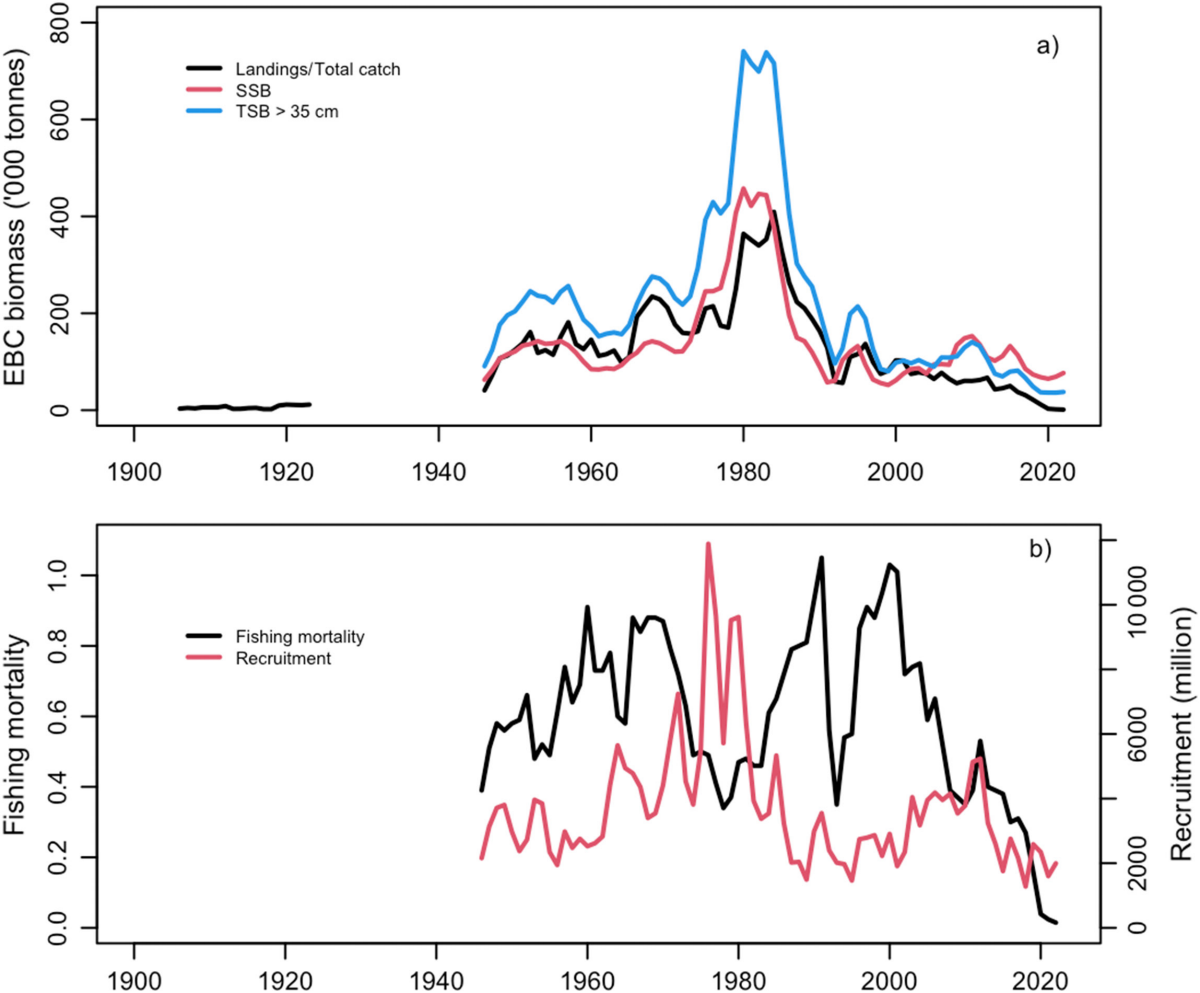
Population metrics of the Eastern Baltic cod (ICES 2023): (a) the reported landings of Baltic cod in ICES SD24‐321946–1965, and total catch (including estimates of discards) 1966–2022, total biomass of cod > 35 cm in length (TSB) and the estimated spawning stock biomass (*SSB*) 1946–2022; (b) fishing mortality for age groups 4–6 (*F*_46_) and the recruitment of under‐yearling (0+) cod.

The Eastern Baltic cod stock (EBC; Figure 1), currently occurring mainly east of the island of Bornholm (Figure 2) has gone through dramatic changes in distribution, total biomass, size structure and productivity over the last 80 years (Figure 3; Hammer et al. 2008; Eero et al. 2015, 2023; Svedäng and Hornborg 2014, 2017; Svedäng et al. 2022a; ICES 2023). From being a major European cod stock, with an all‐time high around the end of the 1970s to the beginning of the 1980s, it is since 2019 under a fishing ban. Multiple drivers have so far been identified to have contributed to the negative development. In the late 1980s, oxygen and salinity conditions were deteriorated to such an extent that cod egg development became impossible at two of three major EBC spawning locations (MacKenzie et al. 2000; Köster et al. 2019; Svedäng et al. 2022a), while recruitment still remained relatively high in the last major spawning location around the island of Bornholm (Figure 3). Further declines in EBC productivity since the mid‐1990s have been attributed to decreases in individual growth, body condition and reduced survival rates (Eero et al. 2012, 2015, 2023; Casini et al. 2016, 2021; Svedäng and Hornborg 2014, 2017; Neuenfeldt et al. 2020; Mion et al. 2021; Lindmark et al. 2023). However, it is still not clear what represents the main causation for the development.

Studying longer time series than contemporary stock assessments may offer improved understanding of what represents symptoms versus causes. For example, observations suggest that Fulton's condition factor *CF*, in sensu (Ricker 1975) in EBC was equally low in the 1940s as it is today (Eero et al. 2023), possibly reflecting lower ecosystem productivity compared to the 1970–1980s (e.g., Eero et al. 2011). Eero et al. (2023) also noted that size structure and fishing pressure have become increasingly decoupled since the beginning of the 2000s; as fishing mortality and size distribution decline concurrently, the decline in size is suggested to be more related to reduced individual growth and lower survival rates.

Presently, although extensively studied, the driver(s) behind reduced individual vigour has remained obscure (Brander 2022; Svedäng et al. 2020, 2022a, 2022b). Changes in *CF* or growth (Mion et al. 2021), have been indirectly related to hypoxia (Casini et al. 2016; Karlson et al. 2020) via, for instance, benthic prey scarcity or directly affecting the scope for the metabolism of cod (Casini et al. 2021; Brander 2022). Neuenfeldt et al. (2020) noted that energetic uptake (KJ/day) for size groups of cod of 21–25 and 26–30 cm was indeed bell‐shaped over time, that is, low in the mid‐1960s increasing up to the end of 1990s, and thereafter declining. For larger size groups (31–35 and 36–40 cm), the energetic uptakes have monotonously been increasing over time up to the mid‐2010s, mirrored in studies on nitrogen content in EBC otoliths as a proxy on feeding rates (Svedäng et al. 2020). Orio et al. (2019) noted juvenile cod (15–30 cm) occurring at deeper depths in the Baltic Sea over time, suggesting higher exposure to water layers depleted of oxygen. However, such a change could not be evidenced from trawl surveys in the Bornholm Basin, the remaining core area of EBC (Figure 2; Svedäng et al. 2022b). The hypothesis of food scarcity is also contradicted by the fact that oxygen conditions and abundances of benthic animals have remained stable in areas in the southern Baltic Sea inhabited by EBC, that is, above 50 m depth (Svedäng et al. 2022a). Furthermore, Lindmark et al. (2023) found that since the 1990s, covariates between for example, EBC and forage animals such as sprat (*Sprattus sprattus*) and benthic crustaceans (*Saduria entomon*), temperature and oxygen had low explanatory value to changes in *CF*.

Another potentially related observation for Eastern Baltic cod is dramatic change in size at maturity over time. The midpoint in the maturity ogive (i.e., the observed length at which 50% of the fish have attained sexual maturity, *L*_50_) has decreased from 40 to around 20 cm between the mid‐1990s and beginning of the 2020s (ICES 2023). This decline has been inferred from previous high fishing pressures/exploitation rates (Andersen et al. 2007; Carlson and Stenseth 2008; Vainikka et al. 2009). Andersen et al. (2007) suggested that only an effective fishing ban would halt the evolution towards smaller sizes at maturity. However, while fishing mortality has successively decreased over the last decade to almost nil (Figure 3), *L*_50_ has continued to decline (ICES 2023) just as Fulton's condition factor has dropped (Casini et al. 2016) and the size distribution has become progressively truncated (Svedäng and Hornborg 2017; ICES 2023). Eero et al. (2023) instead proposed that such increasingly early maturation may lead to higher energetic costs and lower survival rates. In other words, changed conditions for maturation may change life‐history patterns reciprocally, that is, early maturation leads to stunting (c.f. Alm 1959). It also has to be recognised that *L*_50_ around 20 cm implies that maturation starts even earlier and thus at even smaller sizes (c.f. Thorpe 2007). This observation excludes mechanisms such as density of prey in the form of forage fishes, calling for further investigations of size at maturity in EBC relative to the overall stock productivity where longer time series may be useful due to the varied conditions of the Baltic Sea and for Eastern Baltic cod since the early 1900s to present (e.g., Österblom et al. 2007; Casini et al. 2008; Svedäng et al. 2022a, 2022b).

### 1.2 Current Understanding of Changes in Size at Maturity

Over the last decades, life‐history changes such as alterations in size and age at maturity have often been attributed to size‐selective fishing mortality, not least in Atlantic cod (e.g., Rijnsdorp 1993; Hutchings 2005; Olsen et al. 2005; Svedäng and Hornborg 2014). In response to this potential threat to fish stock productivity, the concept of fishery‐induced evolution (FIE) has emerged, where drivers for change in life histories in exploited species are described as either ‘fish evolving to grow more slowly to escape a fishing mortality that commences above a threshold body size’ or ‘fish evolving to grow more slowly because they invest more energy into early maturation respond to a selection pressure that is direct on maturation and indirect on growth’ (Heino, Díaz Pauli, and Dieckmann 2015). Nonetheless, FIE has seldom been sufficiently evidenced because changes in life history may also be due to environmentally‐induced phenotypic plasticity and genetic adaptations in varying and unknown proportions (c.f. Stearns 1989, Thorpe 2007, Wright, Millar, and Gibb 2011). Based on quantitative genetic studies, 20% of the variability in size at maturity in cod have a heritable component, similar to other teleosts (Kolstad et al. 2006; Kristjánsson and Arnason 2014), but despite centuries of exploitation, Atlantic cod (*Gadus morhua*) stocks off North America's east coast exhibit genetic stability (Pinsky et al. 2021). Life‐history modifications and their heritability are however difficult to study because the environmental impact on animals in captivity will differ from what they would experience in the wild (Scheiner 1993). Most studies are thus field observations with rare possibilities of achieving pseudo‐controlled conditions (e.g., Wright, Millar, and Gibb 2011; Zhang et al. 2018).

From a purely theoretical perspective, plasticity in maturation patterns can also be analysed using life‐history theory (Beverton 1992 and the references therein). Central to Beverton and Holt (1957, 1959, 1964) is the von Bertalanffy's growth formula (VBGF; Quinn II and Deriso 1999), expressed as follows: Equation (1):

(1) $$L_{i}=L_{\infty i}\left(1-e^{-K\left(t-t_{0}\right)}\right)$$

where *L*_i_ is the expected or average length at time (or age) *t*, *L*_*∞i*_ is the asymptotic average length of year class *i*, *K* is the Brody growth rate coefficient (units are year^−1^) and *t*_0_ is a modelling artefact. Maturation is supposed to occur close to the steepest part of the curve of growth in length, *L*, against time or age, that is, at the inflexion point, where the curve changes from being concave to being convex (e.g., Jensen 1996). The inflexion point is when *L* ≈ 0.67 and weight at *W* ≈ 0.3, regardless of the value of *K*. The relationships between growth and mortality patterns, expressed in terms of the parameters *K* and *L*_∞_ of *VBGF*, age (*T*_m_) and length at maturation, *L*_50_, and adult [natural] mortality rate (*M*), determines life‐span, production levels and reproductive output (Beverton and Holt 1959; Jensen 1996; Prince et al. 2015). Changes in growth potential, that is, *L*_∞_, thus predict changes in *L*_50_, or, feasibly, the other way around. Improved understanding of the maturation process in fishes in a life‐history context thus involves concurrent analysis of the maturation, growth and mortality rate(s) of the studied species—and environmental drivers.

To disentangle genetic effects (heritability) from environmental‐induced variability (phenotypic plasticity) in maturation patterns, the statistical concept of probabilistic maturation reaction norm (PMRN) has been introduced (Heino, Dieckmann, and Godø 2002; Heino, Díaz Pauli, and Dieckmann 2015). This method describes the probability of an average individual maturing as a function of growth, using size‐at‐age observations as proxies for growth. The estimation of PMRN*s* thus removes most growth and demography impacts from maturation schedules and may retain a potentially genetic residual trend. Further, the method assumes that the effects of many environmental variables on maturation are directed through individual growth. Different studies have come to different conclusion, where, for instance, Wright, Millar, and Gibb (2011) concluded that regional selection for early maturing genotypes provided the most parsimonious explanation for the decreases in PMRN midpoints (≈*L*_50_) in North Sea neighbouring cod subpopulations, while other studies found synchronous changes at *L*_50_ in Lake Erie fishes being unrelated to harvest intensity (Zhang et al. 2018; Gíslason, McLaughlin, and Robinson 2021). These partly contradicting findings imply that the intertwined relationships between size at maturity, fishing pressure, energetic status and environmental stressors/ signals such as temperature are complex and call for further investigations. Furthermore, following life‐history theory, trends in *L*_∞_ observed in *EBC* (Svedäng and Hornborg 2014), could initiate changes in *L*_50_ or vice versa. Likewise, prolonged increased levels of natural mortality might lead to heritable alterations in *K* or *Tm*, as suggested by Neuenfeldt et al. (2020) and extracted from modelling (ICES 2023).

Another potential explanatory factor that has been less explored is the influence of changes in food quality rather than quantity, which may also affect metabolism and, hence, maturity patterns in *EBC* (Svedäng et al. 2020; see also Svedäng 1991). This further highlights the need to combine long‐time series with available information on changing growth conditions. The eutrophic status of the Baltic Sea (Andersen et al. 2017) and increased summer temperatures due to climate change have led to intensified cyanobacteria blooms (Kahru et al. 2020). The transfer of essential nutrients may, therefore, have become more limited (Litzow et al. 2006; Snoeijs and Häubner 2014; Gorokhova 2019). Furthermore, Kulatska et al. (2019) suggested that EBC showed a strong diet preference for herring (*Clupea harengus*) over sprat, while stomach inspections revealed increased proportions of sprat and decreased proportion of herring from 1990 to 2013. Such discrepancies—together with the declines in abundances of western and central Baltic herring, truncations of size distribution in herring (ICES 2023) and reduced fat content in herring in the northern part of the Baltic Proper (Rajasilta et al. 2019) indicate altered food‐web conditions. Development in the nutritional value of herring, such as fat content, in the core area of EBC in the southern part of the Baltic Sea is thus of great interest to investigate.

### 1.3 Study Objectives

Understanding changes in maturity patterns are of paramount importance for the understanding of variability in fish stock productivity. Thus, this study aims to compare long‐term variability (potentially up to ~100 years) in size at maturity (*L*_50_) for EBC with indices of cod productivity (e.g., Fulton's condition factor, size structure, yield‐per‐recruit, total stock biomass and landings) and ecosystem conditions (prey abundance and nutritional quality). Our specific objectives were to extract, value and analyse historical information from national archives from 1919 to 1983 with analytical stock assessments results since 1991 to allow for investigating longer time series on growth conditions. We hypothesise that either factors affecting productivity also affect size at maturity, or vice versa, where an attempt is made to also further the understanding on the influence of ecosystem processes and changes ongoing simultaneously in the Baltic Sea food web.

## 2 Material and Methods

### 2.1 Data to Support Life‐History Parameter Estimation

Studied life‐history parameters, their acronyms, descriptions and indices are listed in Table 1.

**TABLE 1:** List of studied life‐history parameters, their acronyms, description and indices.

Life‐history parameter	Acronym	Description	Index on
Maturity ogive	L_50_	Median length at first maturity in cm	Size at maturity in EBC
Herring fat content	FA	Percentage fat in herring muscle, dimensionless	Energetic status in herring
Length Diversity Index	LDI	Shannon‐Wiener diversity, based on length. Low values indicate a homogenous population, dimensionless	Size distribution in EBC
Length at 95th percentile	L_95_	The sample 95th percentile of the length distribution in cm	Size distribution in EBC
Catch of EBC	EBC Catch	Registered nominal landings and discards in tonnes	Productivity in EBC
EBC SSB	SSB	Estimated biomass of both sexes of mature EBC in tonnes	Productivity in EBC
EBC Total Stock Biomass	TSB_> 35_	Estimated total biomass of all fish above 35 cm in tonnes	Size distribution and productivity in EBC
Condition Factor	CF	Fulton's body condition factor, dimensionless	Body status in EBC
Exploitation Rate, F/Z	ER	The ratio between fishing mortality, F and total mortality, Z	Fishing intensity on EBC
Landings Per Recruit	CPR	The ratio between registered landings and the he estimated number of recruits	Yield and productivity in EBC
Central Baltic Herring SSB	CBH_SSB_	Estimated biomass of both sexes of mature CBH in tonnes	Herring abundance
Sprat SSB	SPR_SSB_	Estimated biomass of both sexes of mature sprat in tonnes	Sprat abundance

#### 2.1.1 Data Sources

Archived reports from Swedish historical trawl surveys, which were conducted for various reasons, contain records on Baltic cod between 1919 and 1983. The archived material is permanently located at the Regional State Archives in Gothenburg (https://riksarkivet.se/‐goteborg), where it can be requested. The survey data are available from locations in the Baltic proper (i.e., ICES SD25‐SD29; Figure 2). The archived material contains three sources of information:

- Source 1: Catch records, including information on the date, location, fishing gear, time of fishing, number of fish per length group (usually 1 cm) per species.
- Source 2: Records on individual cod, giving information on the date, fishing method, fishing location, species, fish length and sometimes including details on sex and maturity stage.
- Source 3: Notes written on the envelopes used for storing biological material such as otoliths and scales saved for age reading purposes, providing information on length, location and date of catch, as well as occasional information on weight, sex and maturity stage.

For the latter period between 1991 and 2022, we used ICES DATRAS database (www.ices.dk) as well as published analytical assessment information (ICES 2023).

#### 2.1.2 Indices on Size Distribution Changes

The historical information used for estimation of EBC size‐related parameter values was based on fishing surveys using demersal trawls in ICES SD25‐29 (Figure 2, Table S1a,b). We used the catch data (source 1) collected during the historical trawl surveys for estimating parameter values related to size distributions and abundance per length class 1919–1983 (Table S1a,b), that is, only hauls with complete information on the number at length. Information on BITS trawl surveys between 1991 and 2021 in the first and fourth quarter was collected from the ICES DATRAS database (downloaded from www.ices.dk 2021‐11‐10). Length diversity index, LDI_*i*_, in year *i* was defined by the Shannon‐Wiener diversity of the annual length‐frequency distribution (Probst, Stelzenmüller, and Kraus 2013, and the references therein) for the annual survey catch where *L*_min_ was set to 20 cm: Equation (2):

(2) $${\mathrm{LDI}}_{i}=-\sum_{k=L_{\min}}^{L_{\max}}\frac{N_{\mathrm{LC}}}{N_{i}}\ln\left(\frac{N_{\mathrm{LC}}}{N_{i}}\right)$$

where *N*_LC_ is the number of fish in length class LC and *N*_*i*_ is the total number in year *i*. Further, we estimated the ‘fish length indicator’ used ICES EBC assessments (ICES 2023), that is, length at the 95th percentile of the length distribution (*L*_95_) for fish ≥ 20 cm. *L*_95_ were estimated both from the historical material, and since 1991, from the BITS in the first (*Q*1) and fourth quarter (*Q*4) surveys, except at the beginning of the 1990s, then surveys were conducted only in *Q*1. Our estimates were considered similar to those provided by Eero (2021a).

#### 2.1.3 Maturity

For estimation of parameter values which included information on length, sex and maturation, data were included irrespectively of the fishing method, that is, (sources 2 and 3). We followed changes in maturity ogive (*L*_50_) in *EBC*, that is, the length at which 50% of the population have attained maturity by ICES SD25‐29 for sexes combined according to the procedure at the assessment working group (ICES 2014a, 2019). Due to sex dimorphism, maturity ogives may differ between sexes in cod stocks (Jørgensen 1990; Radtke and Grygiel 2013). Such discrepancies may lead to biased trends in ogive estimates then both sexes are combined, in case the representation of the two sexes in samples are skewed over time. However, since the sex ration in the samples were rather equally distributed over time (Table S2), ogive estimations were based on the two sexes combined in similarity to other studies (c.f. Eero et al. 2023).

The spawning period of *EBC* has historically occurred from March to September (Bagge et al. 1994), where peak spawning has varied between the second and the third quarter of the year (Hessle 1923; MacKenzie et al. 1996; Wieland, Jarre‐Teichmann, and Horbowa 2000). Estimation of maturity ogives can be made during the pre‐spawning period (Tomkiewicz et al. 2002), or in the postspawning period (Saborido‐Rey and Junquera 1998), or, preferably, during the spawning period (Sampedro, Garabana, and Saborido‐Rey 2018). Accordingly, we used records of sexual maturity obtained from the historical surveys between 1919 and 1983 during the spawning period between April and August (Hessle 1923; Table S2).

All notes from the archives used eight‐degree Maier's scale (Maier 1906), also described in Hessle (1923), which is similar to what has been used more lately (ICES 2014a; Radtke and Grygiel 2013; Eero et al. 2023). Identical to the procedures used by Radtke and Grygiel (2013) and Eero et al. (2023), we considered fish assigned to maturity scale 1–2 as immature, while fish within maturity scale 3–7, that is, was judged as mature or maturing in the present calendar year. It should be noted that the division between stage 2 and 3 is easy to recognise and stage 3 is also the phase when the maturation process has started (c.f. Tomkiewicz, Tybjerg, and Jespersen 2003, Vitale, Cardinale, and Svedäng 2005). If the fish were considered mature or only matured at later stage, this would have led to overestimation of size at maturity in comparison with ICES measurements since 1991. At times, the degree of maturity of the analysed fish was given by a range (e.g., 2–3). On such occasions, we used the lowest point of the given range as the maturation status of the analysed fish.

The selectivity of the fishing gear and depth strata are likely to influence the ogive estimation. Most of the Swedish historical trawling surveys were made with small mesh sizes in the cod end (< 30 mm; Hagberg et al. 2003), though we were unable to account for differences in sampling locations between years.

Logistic regression was fitted to the material, using the r‐script ‘FSA’ developed by Ogle, Wheeler, and Dinno (2018) for estimating *L*_50_ for sexes combined: Equation (3):

(3) $$\log\left(\frac{p}{1-p}\right)=\alpha +\beta X$$

where *p* is the proportion of mature fish in the sample, *α* and *β* are the fitted parameters of the logistic regression and *X* is the explanatory variable. Regression results on *L*_50_ were only accepted at *p‐*values < 0.05 for the estimated parameters. The yearly arithmetic mean value of *L*_50_ was calculated based on the available annual estimates on *L*_50_ in SD25‐29 (Table S3). Samples from SD24 (Western Baltic) and SD30 (Bothnian Sea) were included for comparison reasons.

For the period since 1991, estimates on *L*_50_, based on data from BITS*‐Q1* survey, were taken from ICES (2023), whose procedure are essential similar to the abovementioned (ICES 2019). It should be noted that the estimates on *L*_50_ given by ICES are without variance.

#### 2.1.4 Fulton's Condition Factor and LeCren's K Condition Index

We calculated Fulton's condition factor (CF) according to the formula: Equation (4):

(4) $${\mathrm{CF}}_{i}=100\frac{W}{L^{3}}$$

where *W* was in g and *L* in cm, and the exponent set to equal to 3 (Casini et al. 2016; Eero 2021b). Following Eero et al. (2023), we also compared LeCren's *K* condition index, which minimise bias related to fish length, with Fulton's *CF* by year for all estimates from SD25‐29 combined. First, data from all years were pooled to estimate the parameters *a* and *b* of the length–weight relationship for all cod whose length were between 20 and 100 cm: Equation (5):

(5) $$W=a*L^{b}$$

Next, LeCren's condition index *K* was calculated for each individual fish *i* as the ratio between its weight (in g) and the predicted weight of the fish at a given length (in cm) from the length‐weight relationship: Equation (6):

(6) $$K_{i}=\frac{W_{i}}{a*{L_{i}}^{b}}$$

Since the exponent in the weight‐length relationship (Equation 5) was close 3 (*b* = 2.987722, Table S4: summary of linear regression of log(*W*) ~ log(*a*) + b*log(*L*)), the developments of LeCren's *K* and Fulton's *CF* were similar (results not shown), only *CF* was included in further analyses.

Subsequently, the procedure described in ICES (2021) was followed by estimating *CF* for the size interval 41–60 cm in EBC in SD25‐SD29 in the year *i* by using records on weight (*W*) and length (*L*) from the historical surveys between 1937 and 1983, if the number of records > 5 (Table S5). The yearly arithmetic mean value of *CF* was calculated based on the available annual estimates on *CF* in SD25‐29 (Table S5). Samples from SD24 and SD30 were included for comparison reasons. For the period 1991–2020, estimates on *CF*, based on data from BITS*‐Q1* survey, were provided from Eero (2021b).

#### 2.1.5 Available Estimates of Population Parameters

For the period between 1946 and 2021 on EBC stock spawning biomass (*SSB*_EBC_) in tonnes, total stock biomass of cod above 35 cm in total length (*TSB*_> 35_) in tonnes, EBC recruits in numbers at age 0 and fishing mortality for ages 4–6 (*F*_46_), and for the period between 1946 and 1965 on EBC landings and between 1966 and 2021 on total catch (landings and discards) in tonnes. Information on the EBC size structure and abundance was retrieved from the ICES DATRAS database between 1991 and 2022 (downloading on 2021‐11‐09 at www.ices.dk) for all those national surveys using the Baltic International Trawl Survey (BITS) program and standards (ICES 2014b). The BITS data are from the first and fourth quarters of the year (*Q*1 and *Q*4). In most of the coordinated international surveys, a TW3‐trawl has been used. Time series between 1946 and 1965 on landings and total catch from 1966 and onwards, including both landings and discards (*TSB*), spawning stock biomass (*SSB*), fishing mortality (*F*) and the number of recruits (*REC*) were collected from the latest analytical stock assessment (ICES 2023). The discrepancy in the time series on landings/ total catch is considered to be of little importance since discards just amount to a few percent of the total catch at all times.

We used parameter values for *F*_46_ and natural mortality for ages 4–6 (*M*_46_) for the period 1991–2020, routinely estimated in ICES EBC assessments (ICES 2023). For years preceding this period, that is, between 1946 and 1990, *M*_46_ was set to conventional 0.2. Total mortality, *Z*, was estimated by the formula: Equation (7):

(7) $$Z_{46}=F_{46}+M_{46}$$

#### 2.1.6 Available Estimates of Forage Fish Population Parameters

According to Kulatska et al. (2019), herring is the preferred prey by EBC; in the latest studied period 2007–2013 herring constituted around 20% of the diet for the smallest length group 16–25 cm. The impact of herring abundance and quality on EBC growth and productivity was therefore analysed in a correlative study. For forage fish abundance, recent estimates on the stock spawning biomasses of central Baltic herring (*CBH*_SSB_, downloaded from www.ices.dk on 2024‐02‐21) and sprat (*SPR*_SSB_; ICES 2023) were used (sprat total stock biomass was not available). Data on (*FA*) content were retrieved from SGU (Geological Survey of Sweden). The herring samples were regularly collected in October–November at Utlängan at the southeast corner of the County of Blekinge (Figure 2, Bignert et al. 2017). Fat estimates were made on gillnet caught female herring in sizes 17–19 cm. The percentage of extractable lipids in muscle from herring was determined by extraction with a mixture of polar and non‐polar solvents followed by evaporation of the extract to dryness. Throughout the entire study, all analyses were carried out at Umeå and Stockholm University as parts of the national monitoring of dioxins, chlorinated pesticides, PCBs and brominated compounds in biota (Wiberg et al. 1998). A comparison of two extraction methods used in the monitoring based on herring data from 2016 to 2020 showed that the average lipid concentrations obtained by using ACHEXHEXE and ACHEXDEE extraction (EEA Data Library 4.2.4) were only marginally different.

### 2.2 Data Processing

#### 2.2.1 Indices on Stock Productivity

Catch‐per‐recruit (*CPR*_*i*_) in year *i* was estimated by dividing *EBC* total catch (since 1966 landings and discards, before 1966 just information about landings are available; discards 2%–5% of total catches) in tonnes by the estimated number of age 0 recruits (*REC*) taken from ICES latest assessment (ICES 2023) in year *i*−4, that is, assuming that recruitment preceding the recorded year of total catch was by 4 years (as the weighed midpoint in the yield curve by age for a single cohort, see ICES 2022; table 2.1.9. Eastern Baltic cod in SDs 24–32. Catch‐at‐age): Equation (8):

(8) $${\mathrm{CPR}}_{i}=\frac{\text{Catch}}{{\mathrm{REC}}_{i-4}}$$

Exploitation rate (ER_*i*_) in year *i* was defined as the ratio between *F*_46_ and *Z*_46_: Equation (9):

(9) $${\mathrm{ER}}_{i}=\frac{F_{46}}{Z_{46}}$$

### 2.3 Statistical Analysis

Auto‐correlations were assumed to be present in all the studied population dynamic time series. Therefore, we applied a modified version of the non‐parametric Mann‐Kendall test for evaluating unidirectional trends available in the r‐script bbsmk(), ‘modifiedmk’ package version 1.6 (Patakamuri and O'Brian 2021). Block length was predetermined to 3 years, as longer periods were considered of less population dynamic importance. We followed the recommendation of 2000 bootstrap replicates (Patakamuri and O'Brian 2021 and the references therein). Correlations between studied time series were tested by using the non‐parametric Spearman rho statistic, available in the r‐script package ‘Hmisc version 4.6’ (Harrell Jr and Dupont 2021).

## 3 Results

### 3.1 Length at Maturity and Fulton's Condition Factor

**FIGURE 4:**
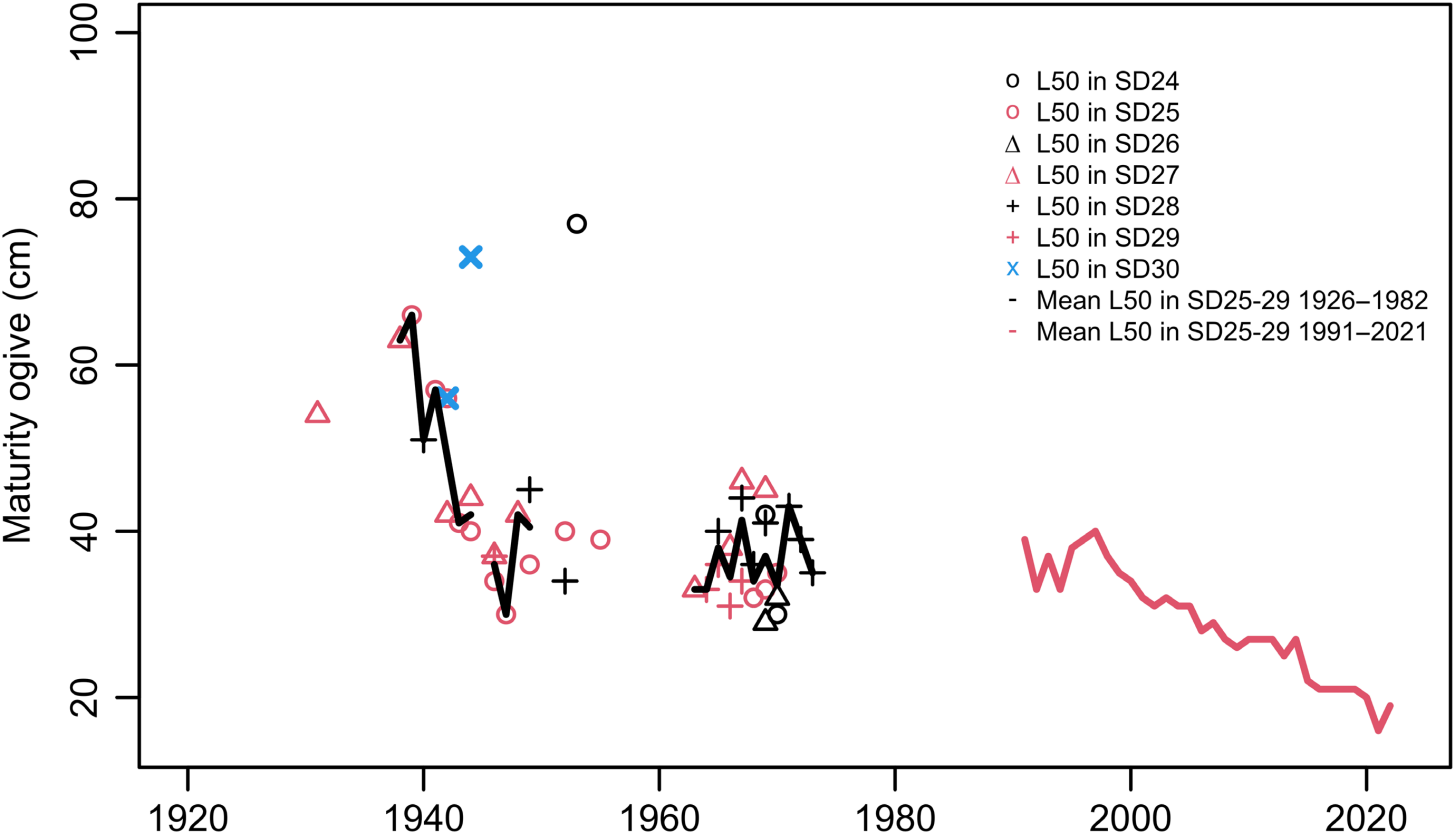
The maturity ogive, *L*_50_, is shown by ICES subdivision (SD) up to 1982. Parameter values for before 1991 are based on Swedish historical notes. The yearly arithmetic mean value of *L*_50_ was calculated from the available annual estimates of *L*_50_ in SD25‐29 (Table S2). Values on *L*_50_ from 1991 to 2021 are estimated for SD25‐29 Baltic Sea (ICES 2023). Samples from SD24 (Western Baltic) probably represent a genetically different stock, and samples from SD30 (Bothnian Sea in the Gulf of Bothnia) represent fish grown under dissimilar feeding and hydrographic conditions.

The data here presented have come from two different sources: (a) from Swedish historical trawl surveys contain records on Baltic cod between 1919 and 1983, and (b) from the ICES DATRAS database between 1991 and 2021. Mean *L*_50_ for SD25‐29 from 1931 to 1983 show initially a sharp decline from around 60 cm to around 40 cm (Figure 4; Mann–Kendall test: *Z*‐Value: −3.2, Sen's Slope: −0.80, *p*‐value < 0.002, Kendall's Tau: −0.45). Adding estimates from ICES (2023) between 1991 and 2022, showed that *L*_50_ still was around 40 cm in the beginning of the 1990s but then fall to the lowest level hitherto recorded (the entire time series: Mann‐Kendall test: *Z*‐Value: −8.1, Sen's Slope: −0.51, *p*‐value < 0.0001, Kendall's Tau: −0.74). The variation was small between subdivisions from the core distribution area of EBC, that is, SD25‐29, whereas estimates from SD24 (Western Baltic) and SD30 (Bothnian Sea) departed more from the mean *L*_50_ for SD25‐29.

**FIGURE 5:**
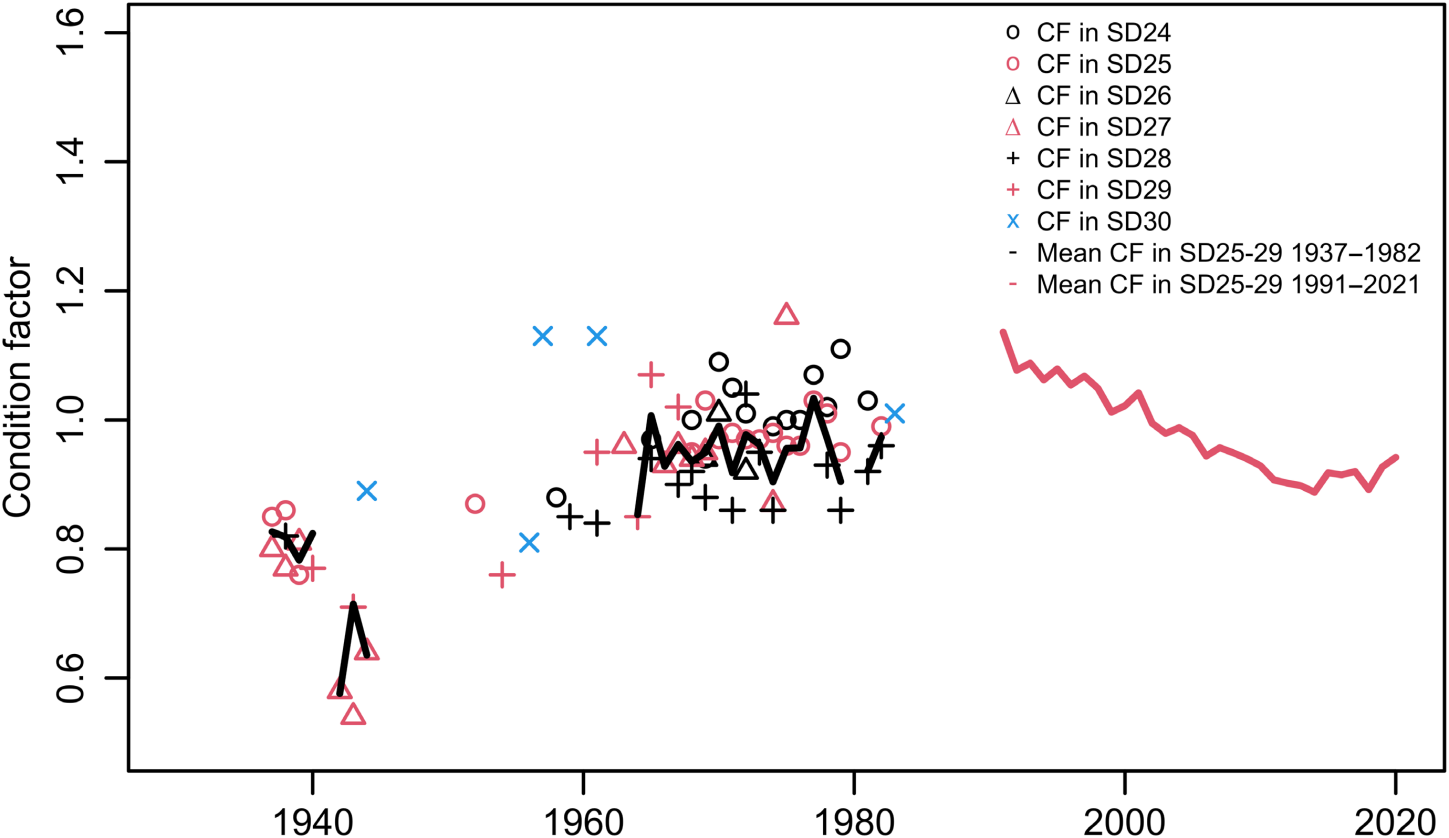
The Fulton's condition factor, *CF*, is shown by ICES subdivision (SD) up to 1982. The yearly arithmetic mean value of *CF* was calculated based on the available annual estimates of *CF* in SD25‐29 (Table S3). Mean values of *CF* from 1991 to 2020 were estimated for the entire EBC (Eero 2021b). Samples from SD24 (Western Baltic) probably represent a genetically different stock, and samples from SD30 (Bothnian Sea in the Gulf of Bothnia) represent fish grown under dissimilar feeding and hydrographic conditions.

Fulton's condition factor, *CF*, was stable from the 1930s up to the 1980s (Figure 5; Mann–Kendall test: *Z*‐Value: 4.0, Sen's Slope: 0.01, *p*‐value < 0.0001, Kendall's Tau: 0.53). Adding the time period 1991–2020 (Eero 2021b), finds a similar decline in *CF* as in *L*_50_, although the level since 1991 has remained higher compared to most of the estimates 1937–1983. Similar to the estimates of *L*_50_, the variation in *CF* between subdivisions in SD25‐29 is rather small, while some estimates of *CF* in SD30 are higher than in SD25‐29.

### 3.2 Comparison of Studied Population Parameters

**FIGURE 6:**
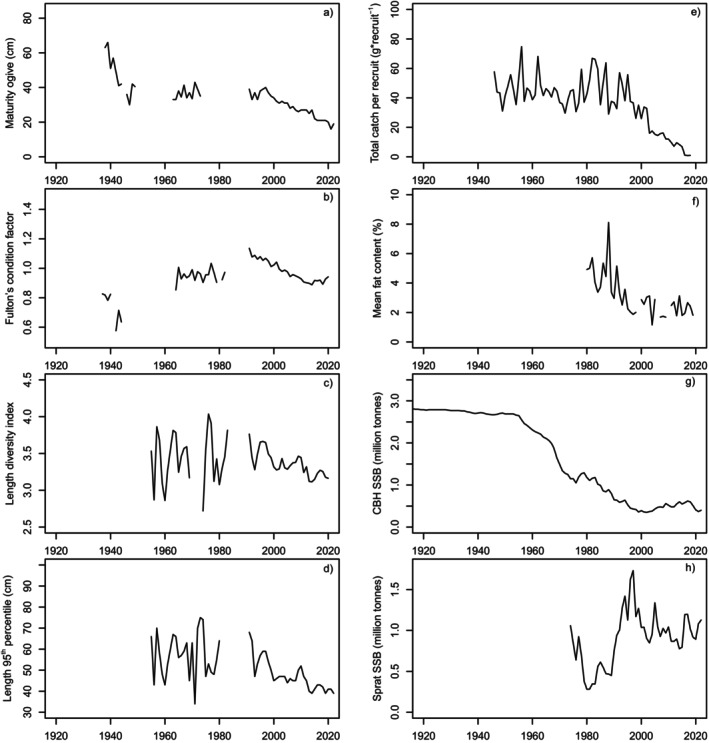
The time series of studied population dynamic variables in SD25‐29 (Figure 2): (a) The maturity ogive, *L*_50_, (cm); (b) Fulton's condition factor, *CF*; (c) Length diversity index, *LDI*; (d) Length of the 95th percentile (cm), *L*_95_; (e) Catch per recruit, *CPR*, that is, until 1965 just landings, since 1966 landings and discards divided by estimated number of age 0 recruits shifted 4 years forward (g * recruit‐1), (f) Fat content in central Baltic herring (*FA*); (g) central Baltic herring spawning stock biomass (*SSB*_CBH_, in tonnes), (h) sprat stock biomass (*SSB*_SPR_, in tonnes). Parameter values for panels a–d before 1991 are based on Swedish historical notes (Table S1a,b, Tables S2 and S3). Since 1991, information has been extracted from ICES (2023) for panel a, for panel b from Eero (2021b), the ICES database DATRAS for panels c‐ and for panel d from Eero (2021a). For panels g–h, the entire time series are taken from ICES (2023).

**FIGURE 7:**
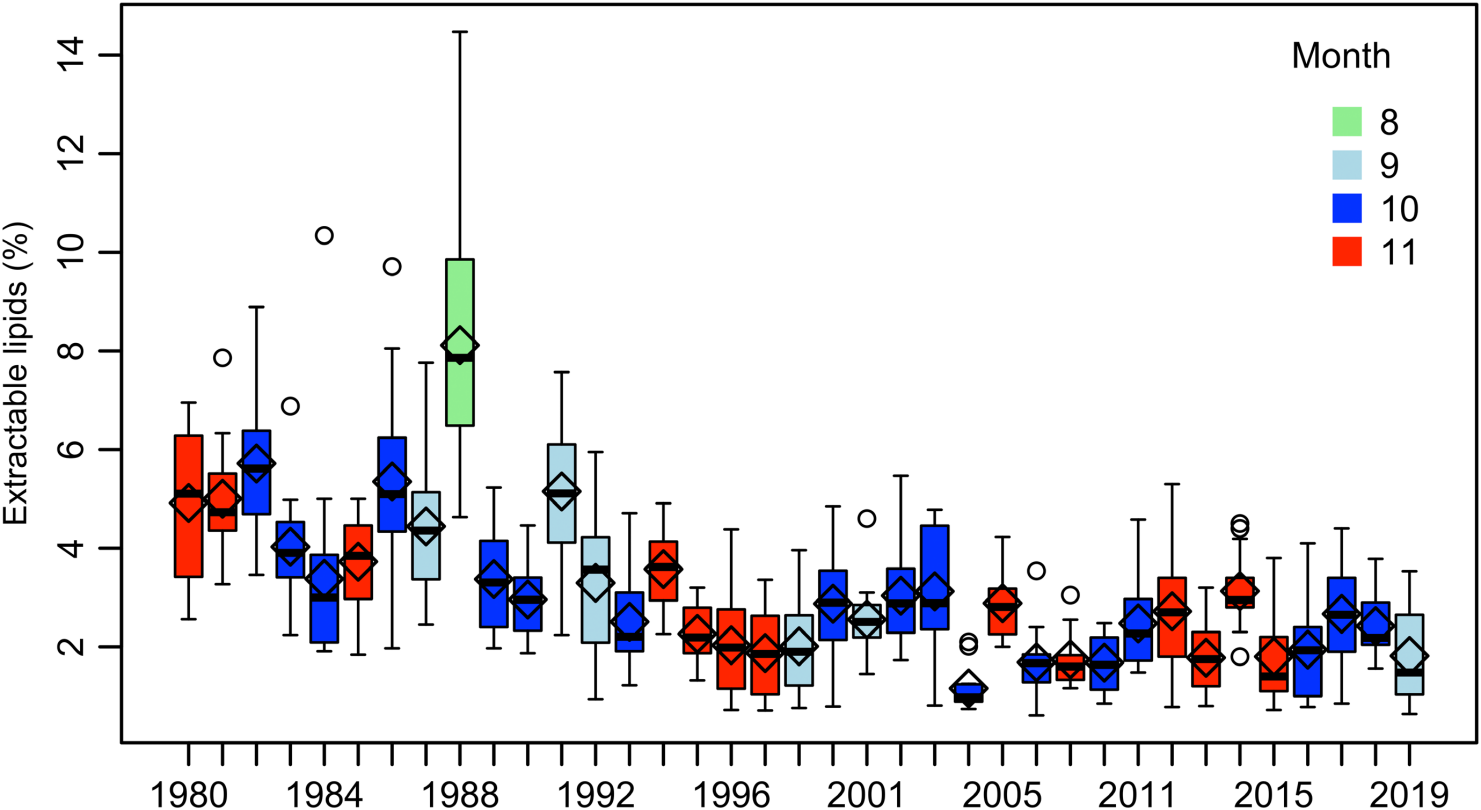
Herring fat content (*FA*) in central Baltic herring (CBH) was estimated from samples taken at Utlängan in southeast Sweden (see Figure 2).

Figure 3a shows EBC spawning stock biomass, *SSB*, total stock biomass, *TSB*_> 35_ for fish above 35 cm since 1940 and Figure 3b shows recruitment and fishing mortality *F*_46_ (ICES 2023). Figure 6a–e show *L*_50_, *CF*, length diversity index, *LDI*, length of the 95th percentile, *L*_95_, total catch per recruit, *CPR*. The mean herring fat content, *FA*, (body percentage, Figures 6f and 7) at Utlängan in southern Baltic Sea (Figure 2). Figure 6g,h show the development of the main forage fish stocks of EBC, mirrored as the spawning stock biomass of the central Baltic herring (*CBH*_SSB_) and Baltic sprat (*SPR*_SSB_).

Correlation analysis (Table 2: Spearman *rho* and *p*‐values; Table S6: the number of observations) of the studied population parameters showed that *L*_50_ was strongly and positively related to indices of both growth and productivity (*LDI*, *L*_95_, *TSB*_> 35_, total catch, *CPR*), and to abundance of herring *CBH*_SSB_, but just weakly to abundance of sprat *SPR*_SSB_, while no correlation was observed between *L*_50_ and *CF*, *SSB* and herring fat content, *FA* (Table 2: Spearman *rho* and *p*‐values; Table S5: the number of observations). A positive correlation was noted between *L*_50_ and exploitation rate, *ER*, suggesting no functional relationship between these two parameters.

**TABLE 2:** Studied life history and population dynamic parameters, (a) Spearman *rho* correlation matrix; (b) *p*‐values.

	Year	L_50_	CF	LDI	L_95_	Catch	SSB	TSB_>35_	CPR	ER	CBH_SSB_	SPR_SSB_	FA
(a)													
Year	1.00	−0.88	0.32	−0.35	−0.65	0.12	−0.27	−0.57	−0.66	−0.73	−0.98	0.46	−0.74
L_50_	−0.88	1.00	0.04	0.69	0.69	0.79	0.14	0.72	0.91	0.80	0.64	0.36	0.33
CF	0.32	0.04	1.00	0.35	0.23	0.10	−0.27	0.01	0.28	0.08	−0.59	0.44	0.21
LDI	−0.35	0.69	0.35	1.00	0.65	0.35	0.08	0.34	0.32	0.34	0.19	0.16	0.14
L_95_	−0.65	0.69	0.23	0.65	1.00	0.58	0.24	0.60	0.58	0.59	0.47	0.10	0.30
Catch	0.12	0.79	0.10	0.35	0.58	1.00	0.69	0.95	0.67	0.75	−0.16	−0.58	0.70
SSB	−0.27	0.14	−0.27	0.08	0.24	0.69	1.00	0.83	0.20	0.22	0.36	−0.62	0.41
TSB_> 35_	−0.57	0.72	0.01	0.34	0.60	0.95	0.83	1.00	0.55	0.62	0.55	−0.57	0.63
CPR	−0.66	0.91	0.28	0.32	0.58	0.67	0.20	0.55	1.00	0.79	0.52	−0.22	0.61
ER	−0.73	0.80	0.08	0.34	0.59	0.75	0.22	0.62	0.79	1.00	0.63	−0.47	0.68
CBH_SSB_	−0.98	0.64	−0.59	0.19	0.47	−0.16	0.36	0.55	0.52	0.63	1.00	−0.60	0.67
SPR_SSB_	0.46	0.36	0.44	0.16	0.10	−0.58	−0.62	−0.57	−0.22	−0.47	−0.60	1.00	−0.58
FA	−0.74	0.33	0.21	0.14	0.30	0.70	0.41	0.63	0.61	0.68	0.67	−0.58	1.00
(b)													
Year	NA	0.0000	0.0147	0.0066	0.0000	0.2625	0.0156	0.0000	0.0000	0.0000	0.0000	0.0010	0.0000
L_50_	0.0000	NA	0.7879	0.0000	0.0000	0.0000	0.3410	0.0000	0.0000	0.0000	0.0000	0.0413	0.1026
CF	0.0147	0.7879	NA	0.0144	0.1049	0.4643	0.0511	0.9612	0.0461	0.5517	0.0000	0.0055	0.2934
LDI	0.0066	0.0000	0.0144	NA	0.0000	0.0075	0.5669	0.0096	0.0154	0.0090	0.1516	0.3367	0.4680
L_95_	0.0000	0.0000	0.1049	0.0000	NA	0.0000	0.0669	0.0000	0.0000	0.0000	0.0002	0.5447	0.1304
Catch	0.2625	0.0000	0.4643	0.0075	0.0000	NA	0.0000	0.0000	0.0000	0.0000	0.1246	0.0000	0.0000
SSB	0.0156	0.3410	0.0511	0.5669	0.0669	0.0000	NA	0.0000	0.0900	0.0494	0.0012	0.0000	0.0108
TSB_> 35_	0.0000	0.0000	0.9612	0.0096	0.0000	0.0000	0.0000	NA	0.0000	0.0000	0.0000	0.0000	0.0000
CPR	0.0000	0.0000	0.0461	0.0154	0.0000	0.0000	0.0900	0.0000	NA	0.0000	0.0000	0.1328	0.0001
ER	0.0000	0.0000	0.5517	0.0090	0.0000	0.0000	0.0494	0.0000	0.0000	NA	0.0000	0.0007	0.0000
CBH_SSB_	0.0000	0.0000	0.0000	0.1516	0.0002	0.1246	0.0012	0.0000	0.0000	0.0000	NA	0.0000	0.0000
SPR_SSB_	0.0010	0.0413	0.0055	0.3367	0.5447	0.0000	0.0000	0.0000	0.1328	0.0007	0.0000	NA	0.0001
FA	0.0000	0.1026	0.2934	0.4680	0.1304	0.0000	0.0108	0.0000	0.0001	0.0000	0.0000	0.0001	NA

## 4 Discussion

### 4.1 Corresponding Changes in Productivity and Maturation Patterns

This study has shown that length at maturity *L*_50_ in EBC has conspicuously declined from above 60 cm to around 20 cm between the 1930s and the 2010–2020s, but it has not been a steady decrease—a plateau is seen between the 1940s and the beginning of the 1990s as reported by ICES (2023). Plausible explanations include observed correlations, where positive correlation exist with productivity and growth‐related indices (*LDI*, *L*_95_, *TSB*_> 35_, total catch, *CPR*) but not with the indices *CF*, *SSB*, *FA* and very weakly to *SPR*_SSB_. The decoupling between *L*_50_ and *SSB* stands in contrast to the strong correlation to *TSB*_> 35_. It may have occurred because *SSB* has not declined in concert with lower growth, as the increase in number of small‐sized mature cod has compensated for the reduction in mean weight (Svedäng et al. 2022a). The unrelated development of *CF* and *L*_50_ is similar to the findings presented by Eero et al. (2023), suggesting that *CF* is an inadequate descriptor of growth potential. Furthermore, *CF* in the 1940s was at even lower levels than it has been in recent decades, while neither the size structure, *L*_50_, nor the productivity (*CPR* or total catch) were as depreciated in the 1940s as it is at present. We also found that the negative developments of *L*_50_ and size‐ and productivity‐related indices were in parallel with the development of *CBH*_SSB_ while not with the trend in *FA* and weakly to *SPR*_SSB_, possibly supporting the view that herring may be more important forage fish for EBC than sprat (Kulatska et al. 2019).

The positive correlation between *L*_50_ and *ER* suggests that when *L*_50_ becomes low, exploitation rate *ER* declines as most fish are found below minimum legal size and are caught only to a minor extent in the cod demersal fishery (ICES 2023). Since *ER* declines in parallel with the size‐related indices, it is rather a nonsense correlation than a causality and underlines that driver behind the truncation of the size distribution is due to lower growth (Svedäng and Hornborg 2017). Reduced growth in *EBC* since the mid‐1990s has also been confirmed by tagging experiments (Mion et al. 2021). Of note the decline in growth found in this study is the opposite of the notion that faster growth at an early age tends to lead to earlier maturation both in the wild and in reared cod (c.f. Hüssy, Eero, and Radtke 2018). However, sharp reductions in growth rate may induce precocious maturation, as suggested by Alm (1959) and experimentally corroborated by Svedäng (1991) in Arctic char (*Salvelinus alpinus*) by enforcing feeding limitations.

### 4.2 Life‐History Changes May Imply Both Phenotypic and Genetic Developments

The strong relationship between *L*_50_ and most size‐related indices and to herring abundance over the last century, suggests that phenotypic plasticity plays an essential part in the variations in the EBC maturation patterns, contrary to proposed evolutionary responses in Atlantic cod (Hutchings 2009) and Baltic cod (Andersen et al. 2007; Vainikka et al. 2009)—or that the decline in size at maturity due to genetically mediated responses leads to lower growth as proposed by Eero et al. (2023). The most parsimonious explanation seems therefore to be that variation in the asymptotic average length (*L*_*∞*_, c.f. Svedäng and Hornborg 2014) regulates *L*_50_ in *EBC*.

It is not straightforward to disentangle drivers and symptoms for *L*_50_ as many parameters are interlinked. Jensen (1996) suggested that the Beverton and Holt life‐history invariants, that is, *L*_50_/*L*_∞_, *K*/*M* and M*T_m_ (where *K* is Brody's growth constant, *M* is the instantaneous rate of natural mortality and *T*_m_ is the age at maturity), reflect fundamental ecological relationships. Variation in *L*_∞_, here approximately indicated by *L*_95_, determines the individual growth potential (Quinn II and Deriso 1999) and may affect size at maturity (*L*_50_) since maturation is supposed to occur close to the inflexion point. Thus, the present decline in individual growth in EBC (Mion et al. 2021) could be a driving factor behind the negative trend in *L*_50_. However, although alterations in *L*_∞_ may not necessarily lead to heritable changes of *L*_50_ but rather reflect the present growth conditions, while also supposedly altering *K*—and thereby affect *L*_50_, through selection (see Equation 1). A possible increase in *Z* over the last three decades, primarily related to an increase in natural mortality, *M*, could thus have long‐term, detrimental consequences for the productivity of EBC if such changes in life‐history traits were inherited. The possibilities of modifications in several life‐history parameters, including that the life‐history invariants are somewhat variable (Prince et al. 2015), suggest that phenotypic plasticity and heredity may be linked to the observed changes in *L*_50_. Thus, the elevated level of *M* over the last decades (ICES 2023) renders caution, as it could have caused adaptive, evolutionary changes.

It is still obscure why growth and survival have declined since the 1990s. Hypoxia in the bottom water layers of the Baltic Sea (Casini et al. 2021) or deterioration in food quality (Svedäng et al. 2020) have been suggested as driving factors. Lower herring abundance and herring fat content cannot be excluded as potential drivers behind low EBC productivity. Even if *L*_50_ and the proxy for yield‐per‐recruit (*CPR*) make good candidates for evaluating stock status for EBC, and *FA* might be a good proxy for external ecosystem pressures, further investigations are needed. With maturation in EBC occurring at *L*_50_ ≈ 20 cm, the maturation process may have commenced just as cod are becoming fish‐eating (Bagge et al. 1994; Kulatska et al. 2019). Thus, investigating the nutrient value of benthic prey and its effect on cod is needed, since it has not declined in abundance in the southern Baltic Sea but may have deteriorated in nutrition value (Svedäng et al. 2022a, 2022b). Such developments probably reflect fundamental ecosystem changes at the primary production level in the Baltic Sea (c.f. Kahru et al. 2020), supported by reductions in individual size in Baltic key species over the last decades such as blue mussels (*Mytilus edulis*) (Liénart et al. 2020) and herring (ICES 2023) as well as in drastic declines in abundances of the benthic amphipod *Monoporeia affinis* (Wiklund, Sundelin, and Rosa 2008) and the seabird eider (*Somateria mollissima*; Ekroos et al. 2012). Changes related to the transfer of energy and matter from primary producers to higher trophic levels seem to be a shared cause.

Supporting this interpretation of the on‐going trophic changes in the Baltic Sea, Steinkopf et al. (2024) found trophic lengthening because of increased cyanobacteria blooms, which, in turn, were due to higher surface temperatures and eutrophication, may have impeded *EBC* stock productivity. Although over‐stating the importance of trophic lengthening, since the decline of *EBC* in the 1980s was merely an effect of hypoxia in deeper layer, hindering cod reproduction in two out of three spawning areas (Svedäng et al. 2022a) and the individual growth was not reduced before but after the 1990s, it is a compelling argument that trophic lengthening of the food chain and hence changes in the energy transfer may have altered the zooplankton community, resulting in reduced mean weight‐at‐age of herring and sprat since the 1990s (ICES 2023).

This development may have eventually affected *EBC* productivity by reducing individual growth already on the juvenile stage, which, in turn, has initiated early maturation (c.f. Svedäng 1991, Thorpe 2007).

## 5 Conclusions

By analysing time series on size at maturity (*L*_50_) relative to growth‐related indices (such as *L*_95_ and *LDI*), condition factor (*CF*) and feeding conditions (*CBH*_SSB_, *SPR*_SSB_ and *FA*), we find that larger sizes at maturity are correlated to good growth conditions, high abundance of large fish and increased yields (*CPR*). Although size at maturity tends to decline on a centurial scale, which might reflect a genetically mediated change, changes in growth conditions constitute a sufficient explanation of alterations in life history. The present extremely early maturation in EBC (*L*_50_ ≈ 20 cm) probably reflects changes at lower trophic levels in the Baltic Sea, leading to discontinued growth and enforced initiation of the maturation process.

## Author Contributions

**Henrik Svedäng:** conceptualization (equal), data curation (equal), formal analysis (equal), funding acquisition (equal), investigation (equal), methodology (equal), project administration (equal), visualization (equal), writing – original draft (equal), writing – review and editing (equal). **Sara Hornborg:** conceptualization (equal), investigation (equal), writing – original draft (equal), writing – review and editing (equal). **Anders Grimvall:** data curation (equal), formal analysis (equal), visualization (equal), writing – review and editing (equal).

## Ethics Statement

The fish data used in this study were partly collected during historical fishery surveys, partly during ordinary field monitoring under the EU Data Collection Framework, for which no specific research or animal ethics permissions are required.

## Conflicts of Interest

The authors declare no conflicts of interest.

## Supporting information


Data S1.

## Data Availability

Data have been retrieved from ICES database (www.ices.dk). The archived material is permanently located at the Regional State Archives in Gothenburg (https://riksarkivet.se/goteborg). Data used in this study can be retrieved from Svedäng, Hornborg, and Grimvall (2024). Centurial life history parameters in Baltic cod (*Gadus morhua*) [Dataset]. Dryad: https://doi.org/10.5061/dryad.4tmpg4fkt.
