# Supplementary material for: Centurial Variation in Size at Maturity of Eastern Baltic Cod (Gadus morhua) Mirrors Conditions for Growth
Source: Ecol Evol. 2024 Oct 13;14(10):e70382. doi: 10.1002/ece3.70382 (PMC11471802; doi:10.1002/ece3.70382)
Supplement: Supplementary file 1 — Data S1. [file ECE3-14-e70382-s001.docx]

Table S1a) The number of years of investigation, the total number of hauls, and the number of caught fish per ICES subdivision (SD).

|  | SD24 | SD 25 | SD 26 | SD 27 | SD 28 | SD 29 | SD30 | Grand total |
| --- | --- | --- | --- | --- | --- | --- | --- | --- |
| Years of investigation | 29 | 28 | 8 | 7 | 19 | 6 | 7 |  |
| Number of hauls | 265 | 416 | 26 | 16 | 70 | 14 | 7 | 836 |
| Number of caught cod | 182185 | 535187 | 5625 | 710 | 49300 | 273 | 1062 | 774342 |

Table S1b) The number of hauls per ICES subdivision (SD) and year.

| Year | SD24 |  | SD25 |  | SD26 |  | SD27 |  | SD28 |  | SD29 |  | SD30 |  | Total |  |
| --- | --- | --- | --- | --- | --- | --- | --- | --- | --- | --- | --- | --- | --- | --- | --- | --- |
|  | No hauls | No cod | No hauls | No cod | No hauls | No cod | No hauls | No cod | No hauls | No cod | No hauls | No cod | No hauls | No cod | No hauls | No cod |
| 1928 | 1 | 22 |  |  |  |  |  |  | 3 | 3 |  |  |  |  | 4 | 25 |
| 1930 | 3 | 22 | 1 | 30 | 1 | 1 |  |  | 1 | 7 |  |  |  |  | 6 | 60 |
| 1933 | 1 | 22 |  |  |  |  |  |  |  |  |  |  |  |  | 1 | 22 |
| 1938 | 6 | 771 | 6 | 160 |  |  |  |  |  |  |  |  |  |  | 12 | 931 |
| 1952 |  | 425 | 2 | 440 |  |  | 4 | 75 | 3 | 732 | 1 | 1 | 1 | 1 | 11 | 1674 |
| 1955 | 12 | 1125 | 14 | 4534 |  |  | 1 |  |  |  |  |  |  |  | 27 | 5659 |
| 1956 |  |  |  |  |  |  | 1 | 98 |  |  |  |  | 2 | 4 | 3 | 102 |
| 1957 | 28 | 3843 | 37 | 4443 | 12 | 2210 | 1 | 2 |  |  |  |  | 10 | 139 | 88 | 10637 |
| 1958 | 3 | 16640 | 6 | 30741 |  |  |  |  |  |  |  |  |  |  | 9 | 47381 |
| 1959 | 2 | 1232 | 7 | 42314 |  |  |  |  | 1 | 2065 |  |  | 7 | 499 | 17 | 46110 |
| 1960 | 6 | 5236 | 17 | 72051 |  |  |  |  | 1 | 1218 |  |  | 7 | 404 | 31 | 78909 |
| 1961 | 7 | 4328 | 15 | 18403 |  |  |  |  | 4 | 3248 |  |  |  |  | 26 | 25979 |
| 1962 | 9 | 9043 | 16 | 12517 | 1 | 286 | 3 | 16 | 6 | 3684 | 6 | 16 |  |  | 41 | 25562 |
| 1963 | 18 | 11087 | 47 | 63020 |  |  | 2 | 453 |  |  | 2 | 46 |  |  | 69 | 74606 |
| 1964 | 11 | 11308 | 41 | 44571 |  |  |  |  |  |  | 2 | 97 | 1 | 1 | 55 | 55977 |
| 1965 | 10 | 13608 | 14 | 19837 |  |  | 3 | 59 | 1 | 874 |  |  |  |  | 28 | 34378 |
| 1966 | 9 | 6226 | 18 | 10699 |  |  |  |  | 2 | 568 |  |  | 1 | 14 | 30 | 17507 |
| 1967 | 26 | 11471 | 31 | 19804 | 1 | 579 |  |  | 7 | 2545 | 1 | 104 |  |  | 66 | 34503 |
| 1968 | 6 | 2740 | 4 | 100 |  |  |  |  | 10 | 3374 | 2 | 9 |  |  | 22 | 6223 |
| 1969 | 3 | 523 | 1 | 10 |  |  |  |  | 1 | 4587 |  |  |  |  | 5 | 5120 |
| 1972 | 8 | 4837 | 13 | 7901 | 8 | 1724 |  |  | 3 | 300 |  |  |  |  | 32 | 14762 |
| 1974 | 2 | 2187 | 2 | 7462 |  |  |  |  |  |  |  |  |  |  | 4 | 9649 |
| 1975 |  |  | 3 | 389 | 1 | 30 | 1 | 7 | 1 | 29 |  |  |  |  | 6 | 455 |
| 1976 | 17 | 4722 | 16 | 4832 | 1 | 642 |  |  |  |  |  |  |  |  | 34 | 10196 |
| 1977 | 10 | 14122 | 12 | 6761 | 1 | 153 |  |  |  |  |  |  |  |  | 23 | 21036 |
| 1978 | 12 | 23230 | 42 | 112184 |  |  |  |  | 8 | 8356 |  |  |  |  | 62 | 143770 |
| 1979 | 12 | 7911 | 12 | 13456 |  |  |  |  | 7 | 10141 |  |  |  |  | 31 | 31508 |
| 1980 | 4 | 3080 | 1 | 2294 |  |  |  |  |  |  |  |  |  |  | 5 | 5374 |
| 1981 | 3 | 4251 | 5 | 8851 |  |  |  |  | 2 | 1228 |  |  |  |  | 10 | 14330 |
| 1982 | 20 | 12927 | 17 | 13973 |  |  |  |  | 4 | 2668 |  |  |  |  | 41 | 29568 |
| 1983 | 16 | 5268 | 16 | 13410 |  |  |  |  | 5 | 3673 |  |  |  |  | 37 | 22351 |
| Total | 265 | 182207 | 416 | 535187 | 26 | 5625 | 16 | 710 | 70 | 49300 | 14 | 273 | 29 | 1062 | 836 | 774364 |

Table S2. The number of immature (Im) and maturing (Mat) per year, sex and ICES subdivision.

| Year | SD25 | | | | SD26 | | | | SD27 | | | | SD28 | | | | SD29 | | | | Grand Total |
| --- | --- | --- | --- | --- | --- | --- | --- | --- | --- | --- | --- | --- | --- | --- | --- | --- | --- | --- | --- | --- | --- |
|  | female | | male | | female | | male | | female | | male | | female | | male | | female | | male | |  |
|  | Im | Mat | Im | Mat | Im | Mat | Im | Mat | Im | Mat | Im | Mat | Im | Mat | Im | Mat | Im | Mat | Im | Mat |  |
| 1919 |  | 1 |  |  |  |  |  |  | 13 | 89 | 8 | 24 |  |  |  |  |  |  |  |  | 135 |
| 1920 |  |  |  |  |  |  |  |  |  | 30 |  | 15 |  | 37 |  | 21 |  | 31 | 2 | 8 | 144 |
| 1921 |  |  |  |  |  |  |  |  | 39 | 323 | 30 | 166 |  |  |  |  | 12 | 1 | 5 | 5 | 581 |
| 1924 |  |  |  |  |  |  |  |  |  |  |  |  | 16 |  | 4 |  |  |  |  |  | 20 |
| 1926 | 56 |  | 34 |  |  |  |  |  |  |  |  |  |  |  |  |  |  |  |  |  | 90 |
| 1927 |  |  | 1 |  |  |  |  |  |  |  |  |  |  |  |  |  |  |  |  |  | 1 |
| 1931 |  |  |  |  |  |  |  |  | 83 | 3 | 64 | 4 |  |  |  |  |  |  |  |  | 154 |
| 1932 | 74 | 12 | 56 | 1 |  |  |  |  | 71 | 1 | 43 | 1 | 28 |  | 27 |  |  |  |  |  | 314 |
| 1934 |  |  |  |  |  |  |  |  | 31 | 13 | 27 | 48 | 6 | 1 | 3 |  |  |  |  |  | 129 |
| 1935 |  |  |  |  |  |  |  |  |  |  |  | 1 |  |  |  |  |  |  |  |  | 1 |
| 1936 |  |  |  |  |  |  |  |  |  |  |  |  | 33 | 2 | 17 |  |  |  |  |  | 52 |
| 1937 | 137 | 30 | 124 | 13 |  |  |  |  | 13 | 1 | 7 | 2 | 130 |  | 93 |  |  |  |  |  | 550 |
| 1938 |  |  |  |  |  |  |  |  | 52 | 1 | 57 | 5 | 220 | 12 | 187 | 4 | 14 |  | 9 |  | 561 |
| 1939 | 126 | 71 | 144 | 59 |  |  |  |  |  |  | 1 |  |  |  |  |  |  |  |  |  | 401 |
| 1940 | 92 | 65 | 60 | 24 |  |  |  |  | 31 | 1 | 17 |  | 36 | 15 | 9 | 24 | 23 | 14 | 17 | 48 | 476 |
| 1941 | 42 | 21 | 28 | 8 |  |  |  |  |  |  |  |  |  |  |  |  |  |  |  |  | 99 |
| 1942 | 85 | 33 | 67 | 27 |  |  |  |  | 40 | 63 | 28 | 12 |  |  |  |  | 17 | 26 | 1 | 35 | 434 |
| 1943 | 9 | 8 | 5 | 14 |  |  |  |  | 83 | 7 | 105 | 6 |  |  |  |  | 8 | 4 | 6 | 9 | 264 |
| 1944 | 76 | 40 | 69 | 83 |  |  |  |  | 76 | 157 | 49 | 56 |  |  |  |  |  |  |  |  | 606 |
| 1946 | 77 | 91 | 77 | 119 |  |  |  |  | 52 | 99 | 12 | 41 |  |  |  |  | 3 | 5 | 1 | 6 | 583 |
| 1947 | 13 | 36 | 7 | 22 |  |  |  |  | 22 | 6 | 1 | 24 |  |  |  |  |  |  |  |  | 131 |
| 1948 |  |  |  |  |  |  |  |  | 43 | 71 | 6 | 33 | 17 | 11 | 2 | 19 | 4 | 9 | 1 | 6 | 222 |
| 1949 | 41 | 87 | 26 | 43 |  |  |  |  |  |  |  |  | 24 | 67 | 5 | 13 |  |  |  |  | 306 |
| 1952 | 41 | 25 | 26 | 28 |  |  |  |  |  |  |  |  | 21 | 32 | 11 | 34 |  |  |  |  | 218 |
| 1953 | 253 | 37 | 187 | 75 |  |  |  |  |  |  |  |  |  |  |  |  |  |  |  |  | 552 |
| 1955 | 213 | 130 | 250 | 89 |  |  |  |  |  |  |  |  |  |  |  |  | 78 | 3 | 73 | 5 | 841 |
| 1961 |  |  |  |  |  |  |  |  |  |  |  |  |  |  |  |  | 1 | 20 |  | 35 | 56 |
| 1962 |  |  |  |  |  |  |  |  |  |  |  |  |  |  |  |  | 11 | 39 | 7 | 33 | 90 |
| 1963 |  |  |  |  |  |  |  |  | 84 | 10 | 49 | 11 |  |  |  |  |  |  |  |  | 154 |
| 1964 |  |  |  |  |  |  |  |  |  |  |  |  |  |  |  |  | 3 | 26 | 8 | 73 | 110 |
| 1965 |  |  |  |  |  |  |  |  |  |  |  |  | 152 | 30 | 109 | 11 | 2 | 17 |  | 5 | 326 |
| 1966 |  |  |  |  |  |  |  |  | 9 | 12 | 16 | 2 |  |  |  |  | 6 |  | 7 | 1 | 53 |
| 1967 |  |  |  |  |  |  |  |  | 135 | 71 | 79 | 24 | 153 | 10 | 128 | 12 | 49 | 51 | 32 | 57 | 801 |
| 1968 | 76 | 27 | 58 | 43 |  |  |  |  |  |  |  |  | 70 | 38 | 48 | 20 |  |  |  |  | 380 |
| 1969 | 62 | 35 | 49 | 54 | 9 | 9 | 7 | 18 | 32 | 23 | 15 | 3 | 51 | 19 | 50 | 4 |  |  |  |  | 440 |
| 1970 | 97 | 48 | 56 | 44 | 21 | 57 | 12 | 52 |  |  |  |  |  |  |  |  |  |  |  |  | 387 |
| 1971 | 1 | 7 | 5 | 20 |  |  |  |  |  |  |  |  | 69 | 19 | 56 | 5 |  |  |  |  | 182 |
| 1972 |  |  |  |  |  |  |  |  |  |  |  |  | 61 | 26 | 32 | 25 |  |  |  |  | 144 |
| 1973 | 243 | 67 | 220 | 66 | 5 |  |  |  | 12 |  | 7 |  | 121 | 16 | 111 | 13 |  |  |  |  | 881 |
| 1977 |  |  |  |  |  |  |  |  |  |  |  |  |  |  |  |  | 26 |  | 19 |  | 45 |
| 1979 | 7 | 42 | 4 | 255 |  |  |  |  |  |  |  |  |  |  |  | 3 |  |  |  |  | 311 |
| Total | 1821 | 913 | 1553 | 1087 | 35 | 66 | 19 | 70 | 921 | 981 | 621 | 478 | 1208 | 335 | 892 | 208 | 257 | 246 | 188 | 326 | 12225 |

Table S3. The length maturity ogive modelling results (*L*_50_, and the length percentile 2.5 and 97.5) for fish caught between April and August by ICES subdivision (SD) 24-31 and year. R-square, number of observations and the length interval for each modelling are show.

| *Year* | *Subdivision* | *Sex* | *L*_50_ | *2.50%* | *97.50%* | *r-square* | *No obs.* | Size interval (cm) | *Comment* |
| --- | --- | --- | --- | --- | --- | --- | --- | --- | --- |
| 1919 | 24 | all |  |  |  |  | 33 | 23-39 | All mature |
| 1953 | 24 | all | 77 | 68 | 96 | 0.14 | 1084 | 9-131 |  |
| 1968 | 24 | all | 32 | 30 | 34 | 0.56 | 222 | 13-56 |  |
| 1969 | 24 | all | 42 | 36 | 48 | 0.56 | 100 | 19-71 |  |
| 1970 | 24 | all | 30 | 28 | 32 | 0.53 | 70 | 16-54 |  |
| 1971 | 24 | all |  |  |  |  | 71 | 12-24 | No mature |
| 1926 | 25 | all |  |  |  |  | 90 | 24-58 | No mature |
| 1932 | 25 | all |  |  |  |  | 143 | 19-70 | No or uncertain solution |
| 1937 | 25 | all |  |  |  |  | 304 | 27-96 | No or uncertain solution |
| 1939 | 25 | all | 66 | 63 | 70 | 0.17 | 400 | 20-104 |  |
| 1940 | 25 | all |  |  |  |  | 241 | 25-89 | No or uncertain solution |
| 1941 | 25 | all | 57 | 51 | 65 | 0.24 | 99 | 28-100 |  |
| 1942 | 25 | all | 56 | 53 | 60 | 0.18 | 212 | 20-83 |  |
| 1943 | 25 | all | 41 | 36 | 48 | 0.43 | 36 | 8-87 |  |
| 1944 | 25 | all | 40 | 36 | 44 | 0.17 | 268 | 12-70 |  |
| 1946 | 25 | all | 34 | 33 | 36 | 0.18 | 364 | 23-89 |  |
| 1947 | 25 | all | 30 | 24 | 33 | 0.24 | 78 | 17-53 |  |
| 1949 | 25 | all | 36 | 30 | 40 | 0.14 | 197 | 12-86 |  |
| 1952 | 25 | all | 40 | 38 | 43 | 0.26 | 120 | 17-60 |  |
| 1953 | 25 | all |  |  |  |  | 552 | 13-70 | No or uncertain solution |
| 1955 | 25 | all | 39 | 38 | 41 | 0.44 | 682 | 4-109 |  |
| 1968 | 25 | all | 32 | 30 | 33 | 0.47 | 204 | 11/53 |  |
| 1969 | 25 | all | 33 | 31 | 34 | 0.44 | 200 | 11-69 |  |
| 1970 | 25 | all | 35 | 34 | 37 | 0.39 | 245 | 12-62 |  |
| 1971 | 25 | all |  |  |  |  | 33 | 22-54 | No or uncertain solution |
| 1973 | 25 | all |  |  |  |  | 596 | 11-31 | No or uncertain solution |
| 1979 | 25 | all |  |  |  |  | 308 | 31-78 | No or uncertain solution |
| 1969 | 26 | all | 29 | 17 | 36 | 0.16 | 43 | 15-70 |  |
| 1970 | 26 | all | 32 | 29 | 34 | 0.60 | 142 | 13-69 |  |
| 1920 | 27 | all |  |  |  |  | 45 | 25-49 | All mature |
| 1921 | 27 | all |  |  |  |  | 588 | 21-83 | All young fish mature |
| 1931 | 27 | all | 54 | 50 | 60 | 0.62 | 154 | 20-73 |  |
| 1932 | 27 | all |  |  |  |  | 116 | 22-54 | No mature but one |
| 1934 | 27 | all |  |  |  |  | 119 | 34-94 | No solution |
| 1937 | 27 | all |  |  |  |  | 23 | 29-76 |  |
| 1938 | 27 | all | 63 | 58 | 100 | 0.24 | 115 | 24-65 |  |
| 1940 | 27 | all |  |  |  |  | 49 | 25-47 | No solution |
| 1942 | 27 | all | 42 | 41 | 45 | 0.30 | 143 | 11-72 |  |
| 1943 | 27 | all |  |  |  |  | 201 | 28-80 | No solution |
| 1944 | 27 | all | 44 | 42 | 45 | 0.33 | 338 | 25-81 |  |
| 1946 | 27 | all | 37 | 27 | 42 | 0.08 | 204 | 25-90 |  |
| 1947 | 27 | all |  |  |  | 0 | 53 | 33-73 | No solution |
| 1948 | 27 | all | 42 | 34 | 47 | 0.14 | 153 | 22-99 |  |
| 1963 | 27 | all | 33 | 30 | 37 | 0.39 | 154 | 15-43 |  |
| 1966 | 27 | all | 38 | 31 | 45 | 0.49 | 39 | 15-56 |  |
| 1967 | 27 | all | 46 | 43 | 49 | 0.16 | 309 | 19-70 |  |
| 1969 | 27 | all | 45 | 42 | 50 | 0.19 | 73 | 30-56 |  |
| 1973 | 27 | all |  |  |  |  | 19 | 17-25 | No mature |
| 1920 | 28 | all |  |  |  |  | 58 | 27-53 | All mature |
| 1924 | 28 | all |  |  |  |  | 20 | 23-50 | No mature |
| 1932 | 28 | all |  |  |  |  | 55 | 24-51 | No mature |
| 1934 | 28 | all |  |  |  |  | 10 | 24-56 | No mature but one |
| 1936 | 28 | all |  |  |  |  | 52 | 23-55 | No solution |
| 1937 | 28 | all |  |  |  |  | 223 | 25-76 | No mature |
| 1938 | 28 | all |  |  |  |  | 423 | 20-70 | No solution |
| 1940 | 28 | all | 51 | 45 | 61 | 0.11 | 84 | 31-83 |  |
| 1948 | 28 | all |  |  |  |  | 49 | 33-76 | No solution |
| 1949 | 28 | all | 45 | 42 | 48 | 0.34 | 109 | 28-82 |  |
| 1952 | 28 | all | 34 | 27 | 37 | 0.10 | 98 | 6-62 |  |
| 1965 | 28 | all | 40 | 37 | 42 | 0.65 | 302 | 13-84 |  |
| 1967 | 28 | all | 44 | 38 | 66 | 0.06 | 303 | 19-39 |  |
| 1968 | 28 | all | 36 | 34 | 37 | 0.48 | 176 | 14-65 |  |
| 1969 | 28 | all | 41 | 39 | 44 | 0.47 | 124 | 20-68 |  |
| 1971 | 28 | all | 43 | 38 | 46 | 0.79 | 149 | 23-76 |  |
| 1972 | 28 | all | 39 | 35 | 47 | 0.07 | 144 | 20-49 |  |
| 1973 | 28 | all | 35 | 34 | 37 | 0.27 | 261 | 14-42 |  |
| 1920 | 29 | all |  |  |  |  | 41 | 25-48 | Almost all mature |
| 1921 | 29 | all |  |  |  |  | 23 | 33-83 | No solution |
| 1938 | 29 | all |  |  |  |  | 23 | 24-60 | No mature |
| 1940 | 29 | all |  |  |  |  | 102 | 32-81 | No solution |
| 1942 | 29 | all |  |  |  |  | 79 | 34-80 | No solution |
| 1943 | 29 | all |  |  |  |  | 27 | 47-84 | No solution |
| 1946 | 29 | all | 37 | 22 | 46 | 0.34 | 15 | 26-77 |  |
| 1948 | 29 | all |  |  |  |  | 20 | 35-70 | No solution |
| 1955 | 29 | all |  |  |  |  | 159 | 23-65 | No solution |
| 1961 | 29 | all |  |  |  |  | 56 | 41-68 | Almost all mature |
| 1962 | 29 | all |  |  |  |  | 90 | 39-63 | Uncertain solution |
| 1964 | 29 | all | 33 | 29 | 38 | 0.74 | 110 | 24-94 |  |
| 1965 | 29 | all | 36 | 24 | 42 | 0.74 | 24 | 31-86 | No solution |
| 1966 | 29 | all | 31 | 29.8 | 32.4 | 0.56 | 14 | 22-32 | No solution |
| 1967 | 29 | all | 34 | 33 | 36 | 0.79 | 188 | 14-101 |  |
| 1977 | 29 | all |  |  |  |  | 45 | 15-34 | No mature |
| 1921 | 30 | all |  |  |  |  | 60 | 23-58 | All mature |
| 1927 | 30 | all |  |  |  |  | 9 | 55-87 | Uncertain solution |
| 1939 | 30 | all |  |  |  |  | 8 | 39-74 | Uncertain solution |
| 1940 | 30 | all |  |  |  |  | 8 | 54-66 | No mature |
| 1942 | 30 | all | 56 | 50 | 62 | 0.37 | 82 | 29-70 |  |
| 1944 | 30 | all | 73 | 67 | 80 | 0.16 | 66 | 37-89 |  |
| 1946 | 30 | all |  |  |  |  | 20 | 37-73 | No solution |
| 1956 | 30 | all |  |  |  |  | 111 | 33-103 | No solution |
| 1961 | 30 | all |  |  |  |  | 41 | 55-87 | All mature |
| 1962 | 30 | all |  |  |  |  | 40 | 59-87 | All mature |
| 1967 | 30 | all |  |  |  |  | 7 | 67-130 | No solution |
| 1983 | 30 | all | 65 | 54 | 167 | 0.12 | 73 | 19-69 |  |
| 1921 | 31 | all | 29 | 23 | 33 | 0.40 | 50 | 28-55 |  |
| 1967 | 31 | all |  |  |  |  | 6 | 37-50 | All mature |

Table S4. Summary of linear regression of log(W) ~ log(a) + b*log(L) for all individual cod betwwen 20 and 100 cm.

Residuals:

Min 1Q Median 3Q Max

-2.03441 -0.07979 0.00761 0.09157 1.41220

Coefficients:

Estimate Std. Error t value Pr(>|t|)

a -4.640073 0.013148 -352.9 <2e-16 ***

b 2.987722 0.003644 820.0 <2e-16 ***

---

Signif. codes: 0 ‘***’ 0.001 ‘**’ 0.01 ‘*’ 0.05 ‘.’ 0.1 ‘ ’1

Residual standard error: 0.163 on 18782 degrees of freedom

Multiple R-squared: 0.9728, Adjusted R-squared: 0.9728

F-statistic: 6.724e+05 on 1 and 18782 DF, p-value: < 2.2e-16

Table S5. The mean Fulton’s condition factor (*C.F.*) and number of observations within the size interval 41-60 cm by ICES subdivision (SD) 24-31 and year.

|  | SD24 |  | SD25 |  | SD26 |  | SD27 |  | SD28 |  | SD29 |  | SD30 |  | SD31 |  |  |  |
| --- | --- | --- | --- | --- | --- | --- | --- | --- | --- | --- | --- | --- | --- | --- | --- | --- | --- | --- |
| Year | C.F. | No of obs. | C.F. | No of obs. | C.F. | No of obs. | C.F. | No of obs. | C.F. | No of obs. | C.F. | No of obs. | C.F. | No of obs. | C.F. | No of obs. | Average 25-29 | No obs  25-29 |
| 1937 |  |  | 0.85 | 9 |  |  | 0.80 | 43 |  |  |  |  |  |  |  |  | 0.83 | 52 |
| 1938 |  |  | 0.86 | 16 |  |  | 0.77 | 116 | 0.82 | 14 |  |  |  |  |  |  | 0.82 | 146 |
| 1939 |  |  | 0.76 | 47 |  |  | 0.81 | 25 |  |  |  |  |  |  |  |  | 0.78 | 72 |
| 1940 |  |  | 0.88 | 1 |  |  |  |  |  |  | 0.77 | 13 |  |  |  |  | 0.82 | 14 |
| 1942 |  |  |  |  |  |  | 0.58 | 16 |  |  |  |  |  |  |  |  | 0.58 | 16 |
| 1943 |  |  |  |  |  |  |  |  |  |  | 0.71 | 10 |  |  |  |  | 0.71 | 10 |
| 1944 |  |  |  |  |  |  | 0.64 | 48 |  |  |  |  | 0.89 | 17 |  |  | 0.64 | 48 |
| 1952 |  |  | 0.87 | 37 |  |  |  |  |  |  |  |  |  |  |  |  | 0.87 | 37 |
| 1954 |  |  |  |  |  |  |  |  |  |  | 0.76 | 58 |  |  |  |  | 0.76 | 58 |
| 1959 |  |  |  |  |  |  |  |  | 0.85 | 11 |  |  |  |  |  |  | 0.85 | 11 |
| 1961 |  |  |  |  |  |  |  |  | 0.84 | 46 | 0.95 | 55 | 1.13 | 30 |  |  | 0.90 | 101 |
| 1964 |  |  |  |  |  |  |  |  |  |  | 0.85 | 67 |  |  |  |  | 0.85 | 67 |
| 1965 | 0.97 | 54 |  |  |  |  |  |  | 0.94 | 34 | 1.07 | 10 |  |  |  |  | 1.01 | 44 |
| 1966 |  |  |  |  |  |  | 0.93 | 30 |  |  | 0.93 | 3 |  |  |  |  | 0.93 | 33 |
| 1967 |  |  |  |  |  |  | 0.96 | 103 | 0.90 | 31 | 1.02 | 83 |  |  | 1.07 | 9 | 0.96 | 217 |
| 1968 | 1.00 | 60 | 0.95 | 97 |  |  | 0.94 | 45 | 0.92 | 41 |  |  |  |  |  |  | 0.94 | 183 |
| 1969 | 0.94 | 9 | 1.03 | 53 | 0.94 | 71 | 0.95 | 66 | 0.88 | 27 |  |  |  |  |  |  | 0.95 | 217 |
| 1970 | 1.09 | 33 | 0.97 | 50 | 1.01 | 72 |  |  |  |  |  |  |  |  |  |  | 0.99 | 122 |
| 1971 | 1.05 | 8 | 0.98 | 16 |  |  |  |  | 0.86 | 21 |  |  |  |  |  |  | 0.92 | 37 |
| 1972 | 1.01 | 134 | 0.97 | 247 | 0.92 | 15 |  |  | 1.04 | 13 |  |  |  |  |  |  | 0.98 | 275 |
| 1973 |  |  | 0.97 | 29 |  |  |  |  | 0.95 | 27 |  |  |  |  |  |  | 0.96 | 56 |
| 1974 | 0.99 | 249 | 0.98 | 586 |  |  | 0.87 | 8 | 0.86 | 46 |  |  |  |  |  |  | 0.90 | 640 |
| 1975 | 1.00 | 77 | 0.96 | 269 |  |  |  |  |  |  |  |  |  |  |  |  | 0.96 | 269 |
| 1976 | 1.00 | 369 | 0.96 | 495 |  |  |  |  |  |  |  |  |  |  |  |  | 0.96 | 495 |
| 1977 | 1.07 | 158 | 1.03 | 305 |  |  |  |  |  |  |  |  |  |  |  |  | 1.03 | 305 |
| 1978 | 1.02 | 426 | 1.01 | 675 |  |  |  |  | 0.93 | 466 |  |  |  |  |  |  | 0.97 | 1141 |
| 1979 | 1.11 | 373 | 0.95 | 578 |  |  |  |  | 0.86 | 522 |  |  |  |  |  |  | 0.90 | 1100 |
| 1981 | 1.03 | 119 |  |  |  |  |  |  | 0.92 | 66 |  |  |  |  |  |  | 0.92 | 66 |
| 1982 |  |  | 0.99 | 122 |  |  |  |  | 0.96 | 156 |  |  |  |  |  |  | 0.97 | 278 |
| Mean /  Total no | 1.03 | 2077 | 0.98 | 3632 | 0.96 | 185 | 0.84 | 507 | 0.90 | 1528 | 0.90 | 385 | 1.01 | 148 | 1.07 | 9 | 0.88 | 6110 |

Table S6. Number of observations (n) corresponding to the Spearman *rho* correlation matrix (Table 2).

|  | Year | L_50_ | CF | LDI | L_95_ | Catch | SSB | TSB_>35_ | CPR | ER | CBH_SSB_ | SPR_SSB_ | FA |
| --- | --- | --- | --- | --- | --- | --- | --- | --- | --- | --- | --- | --- | --- |
| Year | 122 | 57 | 59 | 58 | 60 | 95 | 77 | 77 | 73 | 77 | 119 | 49 | 37 |
| L_50_ | 57 | 57 | 47 | 41 | 46 | 49 | 49 | 49 | 45 | 49 | 57 | 32 | 26 |
| CF | 59 | 47 | 59 | 49 | 50 | 52 | 52 | 52 | 52 | 52 | 59 | 38 | 28 |
| LDI | 58 | 41 | 49 | 58 | 55 | 57 | 57 | 57 | 57 | 57 | 58 | 40 | 30 |
| L_95_ | 60 | 46 | 50 | 55 | 60 | 59 | 59 | 59 | 59 | 59 | 60 | 39 | 27 |
| Catch | 95 | 49 | 52 | 57 | 59 | 95 | 77 | 77 | 73 | 77 | 95 | 49 | 37 |
| SSB | 77 | 49 | 52 | 57 | 59 | 77 | 77 | 77 | 73 | 77 | 77 | 49 | 37 |
| TSB_>35_ | 77 | 49 | 52 | 57 | 59 | 77 | 77 | 77 | 73 | 77 | 77 | 49 | 37 |
| CPR | 73 | 45 | 52 | 57 | 59 | 73 | 73 | 73 | 73 | 73 | 73 | 49 | 37 |
| ER | 77 | 49 | 52 | 57 | 59 | 77 | 77 | 77 | 73 | 77 | 77 | 49 | 37 |
| CBH_SSB_ | 119 | 57 | 59 | 58 | 60 | 95 | 77 | 77 | 73 | 77 | 119 | 49 | 37 |
| SPR_SSB_ | 49 | 32 | 38 | 40 | 39 | 49 | 49 | 49 | 49 | 49 | 49 | 49 | 37 |
| FA | 37 | 26 | 28 | 30 | 27 | 37 | 37 | 37 | 37 | 37 | 37 | 37 | 37 |
